# Woman’s Sensibility to Pain

**Published:** 1897-03

**Authors:** 


### Woman's Sensibility to Pain.

In the description by Ottolengui of his investigations concerning the relative ability of men and women to endure pain, he says:

1. Women are less sensitive to pain than men.
2. Sensitiveness is less in early life, increasing to the twenty-fourth year, and decreasing afterward.
3. The more highly-cultivated classes are most sensitive to pain; degenerates are less sensitive, some of them being very obtuse to pain sensations.
4. Sensibility to pain varies between much greater limits in women than in men, the ability to endure pain reaching a limit far beyond the limit in men.
5. General sensibility is greatest in the nineteenth year.
6. General sensibility depends upon the peripheral nerves.
7. The lesser sensibility to pain in woman is an indication of her inferiority to man, as the uncivilized are less sensitive than the civilized, and degenerates less sensitive than normal individuals.
8. Lessened sensibility to pain in woman is related to her greater longevity.—*Centralblatt f. Nerv. u Psych.*, No. 7.

